# Supplementary material for: Prevalence of periodontitis in adolescents: A systematic review protocol
Source: PLoS One. 2025 May 5;20(5):e0321993. doi: 10.1371/journal.pone.0321993 (PMC12052176; doi:10.1371/journal.pone.0321993)
Supplement: S2 Appendix — (DOCX) [file pone.0321993.s002.docx]

**APPENDIX 2**

| **DATABASE** | **QUERY SEARCH** | **RESULTS** |
| --- | --- | --- |
| **PUBMED** | ("prevalence"[MeSH Terms] OR "prevalence"[Title/Abstract] OR "prevalences"[Title/Abstract] OR "Epidemiology"[MeSH Terms] OR "Epidemiology"[Title/Abstract] OR "Epidemiologic"[Title/Abstract] OR "Epidemiological"[Title/Abstract] OR "frequency"[Title/Abstract] OR "frequencies"[Title/Abstract] OR "occurrence"[Title/Abstract] OR "occurrences"[Title/Abstract] OR "Incidence"[Title/Abstract] OR "Incidences"[Title/Abstract] OR "rate"[Title/Abstract] OR "rates"[Title/Abstract] OR "cross sectional studies"[MeSH Terms] OR "cross-sectional"[Title/Abstract] "cross sectional"[Title/Abstract] OR Surveys[Title/Abstract] OR "Survey"[Title/Abstract]) AND ("periodontal"[Title/Abstract] OR "periodontitis" OR "Periodontium"[MeSH Terms] OR "Periodontium"[Title/Abstract]) AND ("Child"[MeSH Terms] OR "Child"[Title/Abstract] OR "Children"[Title/Abstract] OR "Adolescent"[MeSH Terms] OR "Adolescent"[Title/Abstract] OR "Adolescents"[Title/Abstract] OR "Adolescence"[Title/Abstract] OR "Teens"[Title/Abstract] OR "Teen"[Title/Abstract] OR "Teenagers"[Title/Abstract] OR "Teenager"[Title/Abstract] OR "Youth"[Title/Abstract] OR "Youths"[Title/Abstract] OR "adolescences"[Title/Abstract] OR "adolescency"[Title/Abstract] OR "teenage"[Title/Abstract]) | 1,377 |
| **EMBASE** | ('prevalence':ti,ab,kw OR 'prevalences':ti,ab,kw OR 'epidemiology':ti,ab,kw OR 'epidemiologic':ti,ab,kw OR 'epidemiological':ti,ab,kw OR 'frequency':ti,ab,kw OR 'frequencies':ti,ab,kw OR 'occurrence':ti,ab,kw OR 'occurrences':ti,ab,kw OR 'incidence':ti,ab,kw OR 'incidences':ti,ab,kw OR 'rate':ti,ab,kw OR 'rates':ti,ab,kw OR 'cross sectional':ti,ab,kw OR 'cross-sectional':ti,ab,kw OR 'surveys':ti,ab,kw OR 'survey':ti,ab,kw) AND ('periodontal':ti,ab,kw OR 'periodontitis':ti,ab,kw) AND ('child':ti,ab,kw OR 'children':ti,ab,kw OR 'adolescent':ti,ab,kw OR 'adolescents':ti,ab,kw OR 'adolescence':ti,ab,kw OR 'teens':ti,ab,kw OR 'teen':ti,ab,kw OR 'teenagers':ti,ab,kw OR 'teenager':ti,ab,kw OR 'youth':ti,ab,kw OR 'youths':ti,ab,kw OR 'adolescences':ti,ab,kw OR 'adolescency':ti,ab,kw OR 'teenage':ti,ab,kw) | 1,755 |
| **Web of Science** | TS=("prevalence" OR "prevalences" OR "Epidemiology" OR "Epidemiologic" OR "Epidemiological" OR "frequency" OR "frequencies" OR "occurrence" OR "occurrences" OR "Incidence" OR "Incidences" OR "rate" OR "rates" OR "Cross Sectional" OR "Cross-Sectional" OR "Surveys" OR "Survey") AND TS=("periodontal" OR "periodontitis") AND TS=("Child" OR "Children" OR "Adolescent" OR "Adolescents" OR "Adolescence" OR "Teens" OR "Teen" OR "Teenagers" OR "Teenager" OR "Youth" OR "Youths" OR "adolescences" OR "adolescency" OR "teenage") | 2,050 |
| **Lilacs** | (("prevalence" OR "prevalences" OR "Epidemiology" OR "Epidemiologic" OR "Epidemiological" OR "frequency" OR "frequencies" OR "occurrence" OR "occurrences" OR "Incidence" OR "Incidences" OR "rate" OR "rates" OR "Cross Sectional" OR "Cross-Sectional" OR "Surveys" OR "Survey" OR Prevalencia OR Frequência OR Frequências OR Epidemiologia OR  Epidemiológico OR Ocorrência OR Ocorrências OR Incidencia OR "Estudos Transversais" OR Frecuencia OR Frecuencias OR Ocurrencia OR Ocurrencias OR "Estudios Transversales" OR "Estudios de Prevalencia") AND ("periodontal" OR "periodontitis" OR "periodontite") AND ("Child" OR "Children" OR "Adolescent" OR "Adolescents" OR "Adolescence" OR "Teens" OR "Teen" OR "Teenagers" OR "Teenager" OR "Youth" OR "Youths" OR "adolescences" OR "adolescency" OR "teenage" OR Criança OR Crianças OR Niño OR niños OR Adolescente OR Adolescentes OR Adolescencia OR Jovem OR Jovens OR Juventude OR jóvenes)) AND ( db:("LILACS")) | 977 |
| **ProQuest Dissertations & Theses Global (PQDT Global**) | noft("prevalence" OR "prevalences" OR "Epidemiology" OR "Epidemiologic" OR "Epidemiological" OR "frequency" OR "frequencies" OR "occurrence" OR "occurrences" OR "Incidence" OR "Incidences" OR "rate" OR "rates" OR "Cross Sectional" OR "Surveys" OR "Survey") AND noft("periodontal" OR "periodontitis") AND noft("Child" OR "Children" OR "Adolescent" OR "Adolescents" OR "Adolescence" OR "Teens" OR "Teen" OR "Teenagers" OR "Teenager" OR "Youth" OR "Youths" OR "young" OR "youngs" OR "adolescences" OR "adolescency" OR "teenage") | 669 |
| **Google Scholar** | ("periodontal" OR "periodontitis" OR "Periodontales") AND (Child OR Children OR Adolescent OR Criança OR Crianças OR Niño OR niños OR Adolescente OR Adolescentes) AND (prevalence OR frequency OR frequencies OR occurrence OR occurrences OR Incidence OR Incidences OR rates OR "Cross Sectional" OR "Cross-Sectional" OR Prevalencia OR Frequência OR Ocorrência OR Incidencia OR "Estudos Transversais" OR Frecuencia OR Ocurrencia OR "Estudios Transversales" OR "Estudos de Prevalência") | 100 |
