# Supplementary material for: Prevalence of periodontitis in adolescents: A systematic review protocol
Source: PLoS One. 2025 May 5;20(5):e0321993. doi: 10.1371/journal.pone.0321993 (PMC12052176; doi:10.1371/journal.pone.0321993)
Supplement: Attachment — Submitted filename: Response to reviewers.AB.docx [file pone.0321993.s004.docx]

Dear Dr. Emily Chenette
Editor-in-Chief

PLOS ONE

We appreciate the opportunity to revise our article. We have carefully worked on incorporating the reviewers' suggestions to enhance the quality of the manuscript. Text modifications and additions were highlighted in yellow.

**Reviewer #1**

1. *“Please provide as much information as possible. Note that the word maximum is 300. Do not provide meaningless phrases with this section.”*

**Response:** We sincerely appreciate the valuable feedback provided. Based on the suggestions received, we have worked carefully to improve the mentioned section and ensure that the manuscript meets the expected standards. We are confident that the revisions have strengthened the quality of the work. We have added important information in the abstract, considering the methods used and objectives of the research, ensuring that the essential content is presented clearly and objectively, according to the maximum number of words. The modifications were highlighted and they can be found on page 2 and 3, lines 37 to 52, as follows in red:

A search will be conducted in the following databases: EMBASE, LILACS, PubMed/MEDLINE, and Web of Science. The gray literature will also be consulted through Google Scholar and ProQuest. No language or time of publication restrictions will be applied. To determine which studies will be included, two independent reviewers will select the articles through a two-phase process. In the first phase, reviewers will assess the title and abstract, and in the second phase they will read the full text using predefined eligibility criteria. Data collected will include author, study design, year of publication, country, sample size, sex, age, diagnostic methods, definition of periodontitis (including by sex), reliability of criteria, examination protocol, case classification and prevalence rates.

The risk of bias will be assessed according to the JBI Critical Appraisal Checklist for Studies Reporting Prevalence Data. Results will be synthesized into a narrative description and, if possible, a meta-analysis will be conducted.  The systematic review methodology will enable a comprehensive and accurate analysis of the available evidence. Moreover, this approach may contribute to clinical decision-making, promoting the use of more appropriate and preventive treatments. (Page 2 and 3)

2. *"(...) its worldwide prevalence has been defined to be around 60%, (...)." This number would seem too low, depending on the the respective investigations. Please double check and adapt carefully.*

**Response:** We appreciate your insightful comments. The information in question has been thoroughly reviewed. The modifications were highlighted and they can be found on page 2, lines 30 to 32, as follows in red:

Periodontitis is a common periodontal condition in the adult dentate population, affecting approximately 6 in 10 individuals. Among those affected, approximately 25% develop the most severe form of the disease. The prevalence in adolescents is not well established.

Reference: Trindade D, Carvalho R, Machado V, Chambrone L, Mendes JJ, Botelho J. Prevalence of periodontitis in dentate people between 2011 and 2020: A systematic review and meta-analysis of epidemiological studies. J Clin Periodontol. 2023;50(5):604-26.

3. *"No language or time of publication restrictions will be applied." This would seem mandatory, indeed. However, why do you restrict your research to four databases only (EMBASE, LILACS, PubMed/MEDLINE and Web of Science)?”*

**Response:** Thank you for the feedback. We have followed the orientations from the *Cochrane Handbook for Systematic Reviews.* As recommended by the *Handbook*, the search should be conducted in at least three databases. Additionally, gray literature will also be considered.

Reference: Lefebvre C, Glanville J, Briscoe S, Featherstone R, Littlewood A, Metzendorf M-I, Noel-Storr A, Paynter R, Rader T, Thomas J, Wieland LS. Chapter 4: Searching for and selecting studies [last updated September 2024]. In: Higgins JPT, Thomas J, Chandler J, Cumpston M, Li T, Page MJ, Welch VA (editors). *Cochrane Handbook for Systematic Reviews of Interventions* version 6.5. Cochrane, 2024. Available from [www.training.cochrane.org/handbook](https://training.cochrane.org/handbook).

4. *"Two independent authors will select the studies in a two-phase process based on predefined eligibility criteria." This does not correspond to your Meths section; there, you have stated that "The search will be done by the first author (A.B.O.M)."*
5. *"Two independent reviewer’s authors (A.B.O.M. and L.R.H.) will select the studies in two phases." Again, this would seem to contradict "The search will be done by the first author (A.B.O.M)." Please clarify.*

**Response:** We appreciate your feedback. One author will apply the search strategy to the databases. After that, two authors will select the studies in Rayyan software. We tried to clarify this in the text as follows:

The first author (A.B.O.M) will apply the search strategy in each database. (Line 150 and 151, page 7). In the screening software, two independent reviewer’s authors (A.B.O.M. and L.R.H.) will select the studies in two phases. In phase 1, they will evaluate the titles and abstracts according to the eligibility criteria.  After that, in phase 2, they will analyze the full-texts and select articles by the same criteria. Also, they will crosscheck all the results found. If disagreements arise, a third reviewer (J.M.D.O.) will make a final decision on both phases. (Line 159 to 163, page 7 and 8).

6. *Even if considering "(L.R.H.)" as second reviewer AND "(J.M.D.O.)" to resolve possible disagreements, there is a total of 8 (EIGHT!) Co-Authors, but the tasks of the latter would not seem clear. Please clarify.*
7. *Same with the current draft. Please clarify the responsibilities of all 8 Co-Authors.*

**Response:** Thank you for your comment. The authors of this protocol will be the same ones who will develop the systematic review. Each author will play an important role in developing the systematic review. In order to carry out a proper review, a group of experts is needed, both in the research topic and the methodological area. We've added everyone's contribution to the manuscript. We emphasize that all contributions followed the guidelines of the International Committee of Medical Journal Editors (ICMJE), ensuring fair attribution of authorship.

This systematic review protocol was produced, reviewed, and approved by all authors.

A.B.O.M. will work as a first reviewer on study conceptualization, study selection, data collection, data analysis and the initial manuscript draft.

L.R.H. and J.M.D.O. will work as the second and third reviewers, respectively. They will participate in study conceptualization, study selection, data collection, data analysis, and revising the initial manuscript draft.

P.P., C.M.S., G.Z.S., and C.M. will work on study conceptualization, study design, and data analysis. They will review the draft and approve the final manuscript. They compose a team of experts. C.M.S. and G.Z.S. are Periodontists, P.P. is Implantologist, and C.M. background is in Pediatric Dentistry. All are experienced with systematic reviews.

G.D.L.C. will work on study conceptualization, study design, data analysis, draft review and final manuscript approval before submission. She is the team's supervisor and an expert in systematic review methodology. (Line 280 to 293, page 12 and 13)

8. *"What is the worldwide prevalence of periodontitis in adolescents?" While the main question would seem interesting, please provide a sound rationale. From the clinical experience it would seem clear that periodontitis is not a daily issue with adolescents. Please elaborate both aims and objectives more clearly.*

**Response:** Thank you for the feedback received. Based on the valuable observations, we have revised and improved the original text, focusing on the clarity of the objectives and the strengthening of the rationale. The document now presents a more robust rationale, considering clinical experience as well as the risks associated with periodontitis in adolescents, such as early tooth loss and its impact on systemic diseases. The modifications will enhance the content's clarity and suitability regarding the proposed topic, as seen below.

A SR on this topic is important not only for clarifying the overall prevalence of periodontitis among adolescents but also for identifying high-risk groups through the identification of associated factors. This knowledge makes it possible to concentrate efforts on preventing and treating periodontitis for those in most need, which can minimize the progression of the disease in adulthood. Finally, for its global relevance since there are regional and socioeconomic variations in the impact of periodontitis on adolescents and studies on prevalence in different populations can offer valuable insights for public health. (Page 4 and 5, line 93 to 100).

Reference: Hung M, Kelly R, Mohajeri A, Reese L, Badawi S, Frost C, et al. Factors Associated with Periodontitis in Younger Individuals: A Scoping Review. J Clin Med. 2023;12(20).

9. *"(...), being capable of causing discomfort in the gingival region, bone damage and early tooth loss. Clear!" Please double check and revise carefully. What do you want to say when referring to "Clear!"?*

**Response:** We appreciate the valuable contributions that enabled this revision, ensuring that the content meets the required standards. Following a careful analysis of the text and considering the received suggestion, we inform you that the term "clear!" previously mentioned has been removed.

10. *"That is why this study aims to identify the prevalence of this disease in adolescents." Please note that "no available SRs estimating the worldwide prevalence of periodontitis in adolescents" would not seem convincing. Remember that one possible reason for the observed non-availability could be that no researchers did see any sound reasons up to now. Again, clearly and convincingly elaborate why this study would seem mandatory, and discuss.*
11. *"To date, no SR has explored the prevalence of periodontitis in adolescents." Please see comments given above. This would not seem a convincing rationale. Please discuss carefully but thoroughly.*

**Response:** We sincerely appreciate the feedback received. Based on the valuable contributions, I have modified the text according to the response presented to question 8 above (modifications in the text may be found on page 4 and 5, line 93 to 100, highlighted in yellow). We hope the modification will enhance the clarity and reinforce the importance of the topic.

Reference: Associated with Periodontitis in Younger Individuals: A Scoping Review. J Clin Med. 2023;12(20).

12. *Do you have any null hypotheses? Remember that H0 must be deducible from the forgoing thoughts.*

**Response:** We appreciate your feedback. In a systematic review, the use of a null hypothesis (or even a formal hypothesis) is not as common as in empirical or experimental studies. This is because the main goal of a systematic review is to synthesize and critically analyze the existing literature to answer a given research question rather than directly testing a hypothesis through the collection of new data. Besides, since this is a systematic review of prevalence, it is expected to present a more descriptive nature, in contrast to systematic reviews of intervention, to which hypothesis test is expected.

Reference: Cumpston M, Lasserson T, Flemyng E, Page MJ. Capítulo III: Relatando a revisão [última atualização em agosto de 2023]. Em: Higgins JPT, Thomas J, Chandler J, Cumpston M, Li T, Page MJ, Welch VA (editores). *Cochrane Handbook for Systematic Reviews of Interventions* versão 6.5. Cochrane, 2024. Disponível em [www.training.cochrane.org/handbook](http://www.training.cochrane.org/handbook).

13. *"A preliminary search strategy was conducted (...)." This again would seem unclear. Please provide both aims and results of your "preliminary search".*

**Response:** The development of a preliminary search strategy with the assistance of Medical/healthcare librarians and information specialists allows the identification of reference articles on the topic. This endorses the feasibility of the systematic review and helps the review team improve the definitive search strategy. We have clarify it in the text as follows:

A preliminary search strategy was developed with the assistance of a medical/health librarian and information specialists. This search strategy will be adapted for the following databases: EMBASE, LILACS (in Spanish: Literatura Latinoamericana y del Caribe en Ciencias de la Salud), PubMed/MEDLINE, and Web of Science. (Line 143 to 145, page 7).

14. *Please provide the date of your PROSPERO pre-registration.*

**Response:** Thank you for your comment. We have added the number at the end of the abstract and in the methods section, as follows:

This SR protocol was developed following the guidelines of Preferred Reporting Items for Systematic Review and Meta-Analyses Protocols (PRISMA-P) (Appendix 1), and it was registered in the International Prospective Register of Systematic Reviews (PROSPERO) on July 17, 2024, database under number CRD42024566374. (Line 108 to 109, page 5).

15. *Please provide the date of your start of literature research.*

**Response:** This is a protocol for a systematic review. We have already applied the search on January 4, 2024

16. *Do not use legal terms. Delete "TM", "®", and so on.*
17. *“With ALL materials and methodologies (including statistical software), please use general names with your text, followed by (brand name; manufacturer, city, ST[ate - abbreviated, if US], country) in parentheses. Stick to semicolon. Remember that reproducibility must be ensured.”*

**Response:** We appreciate your comments. We apologize; after reviewing and considering your observations, we have made the necessary corrections.

The file from each database will be imported into a reference manager (EndNote-Web; Clarivate, Saint Helier, Jersey) where duplicates will be removed. Subsequently, the references will be imported into a screening software (Rayyan, Qatar Computing Research Institute, Data Analytics, Doha, Qatar). (Line 154 to 157, page 5).

The data will be stored in a data extraction form that will be developed using a spreadsheet program (Microsoft Excel; Microsoft Corporation, Redmond, WA, USA). (Line 169 to 170, page 8).

If quantitative synthesis is appropriate, a proportion and a subgroup meta-analysis will be performed using the statistical computing software (R Statistics version 3.4.4; The R Foundation for Statistical Computing, Vienna, Austria) using the random effect measure and considering the range 95% confidence level from each study was included. (Line 194 to 195, page 9).

18. *Same with "and a second reviewer (L.R.H.) will crosscheck (data from the included studies)". This would mean that ONLY those studies found by A.B.O.M. will be crosschecked, right? What about the studies A.B.O.M. has missed/overlooked/ignored? Who will really control A.B.O.M.'s work?.*

**Response:** The studies will be selected by two authors to reduce the bias. After the decision about inclusion, the data from these included studies will be extracted by A.B.O.M. and cross-checked by LRH. Yet, according to the Cochrane Handbook for Systematic Reviews, the selection of studies by one author is not forbidden.

“Ideally, screening of titles and abstracts to remove irrelevant reports should also be done in duplicate by two people working independently (although it is acceptable that this initial screening of titles and abstracts is undertaken by only one person).”

Reference: Lefebvre C, Glanville J, Briscoe S, Featherstone R, Littlewood A, Metzendorf M-I, Noel-Storr A, Paynter R, Rader T, Thomas J, Wieland LS. Chapter 4: Searching for and selecting studies [last updated September 2024]. In: Higgins JPT, Thomas J, Chandler J, Cumpston M, Li T, Page MJ, Welch VA (editors). *Cochrane Handbook for Systematic Reviews of Interventions* version 6.5. Cochrane, 2024. Available from [www.training.cochrane.org/handbook](https://training.cochrane.org/handbook).

19. *Please note that this section has not been convincingly elaborated, and clearly would seem perfectible*

**Response:** Dear Editor, we sincerely appreciate your observations. Following a thorough reading and analysis, and taking your suggestions into account, we have enhanced the rationale of our work with clearer and more precise justifications. Please see the answer to Question 8 above.

20. *See comments given above. Please provide the aspects of the CURRENT draft. There has been no Data curation, no Formal analysis, and no Investigation up to now.*

**Response:** We sincerely appreciate your observations, which are highly relevant. This manuscript is a systematic review **protocol**, as you can see in ([crd.york.ac.uk/PROSPERO/display_record.php?RecordID=566374](https://www.crd.york.ac.uk/PROSPERO/display_record.php?RecordID=566374)). It aims at outlining the research plan and methodology to be followed in a future study. Given the preparatory nature of this type of manuscript, the stages of Data Curation, Formal Analysis, and Investigation have not yet been carried out, as these phases are scheduled to occur after the protocol's approval and subsequent execution of the study.

**Reviewer #2**

*The protocol demonstrates originality; however, to be deemed acceptable, a thorough revision addressing the points below is necessary:*

*Background: The current manuscript lacks a comprehensive presentation of the relationship between adolescents and periodontitis. I recommend a more thorough exploration of this relationship.*

*Methodology, Results, and Discussion: There is significant confusion in the formatting of the Results and Discussion sections. I request that these sections be reformulated and systematically organized.*

**Response:** We appreciate the constructive feedback and the positive assessment of the choice of topic and the description of the methodology. We have add a paragraph in the introduction about the relationship between adolescents and periodontitits. Regarding the discussion, since this is a protocol and there are no results, the discussion is indeed limited. Nonetheless, we have made an effort to make it more robust by including initial information that can enrich the understanding of the topic.
