# Supplementary material for: Prevalence of periodontitis in adolescents: A systematic review protocol
Source: PLoS One. 2025 May 5;20(5):e0321993. doi: 10.1371/journal.pone.0321993 (PMC12052176; doi:10.1371/journal.pone.0321993)
Supplement: Attachment — Submitted filename: Answer to reviewers.AB.docx [file pone.0321993.s005.docx]

Dear Dr. Emily Chenette
Editor-in-Chief

PLOS ONE

We appreciate the opportunity to revise our article. We have carefully worked on incorporating the reviewers' suggestions to enhance the quality of the manuscript. Text modifications and additions were highlighted in yellow.

**Reviewer #1**:

1. *“With your reply/your revision, you have indicated that "(your study) was registered in the International Prospective Register of Systematic Reviews (PROSPERO) on July 17, 2024 (...)". At the same time, you state that "we have already applied the search on January 4, 2024". Please note that "prospective registration aims to reduce bias in the conduct and reporting of research and to increase transparency; in addition, prospective registration of systematic reviews is argued to help preventing unintended duplication, thereby reducing research waste". See more information at https://systematicreviewsjournal.biomedcentral.com/articles/10.1186/s13643-021-01877-1.You might wish to go to https://pmc.ncbi.nlm.nih.gov/articles/PMC10123738/ for more reasons and information. - Same with https://pubmed.ncbi.nlm.nih.gov/31492940/.”*

**Response:** We appreciate the observation and acknowledge the importance of prospective registration of systematic reviews in enhancing transparency and preventing unintentional duplication, as highlighted. However, we would like to clarify that the initial search was a pilot test, it was conducted as a test to assess the feasibility of the study and refine the strings.

Subsequently, with the assistance of a librarian the final strategy for each database was constructed. Then, the protocol was registered with PROSPERO on July 17, 2024, ensuring that the subsequent stages of the study adhered to the standards of transparency and methodological rigor associated with registration. We remain committed to conducting research responsibly and aligning our processes with best practices whenever possible.

To clarify this, we have changed the text adding this information, as follows:

“The preliminary search strategy was developed and tested on PubMed on January 4, 2024. After the tests, with the assistance of a health sciences librarian and information specialists the final search strategy was developed for each database”.

(Page 7, line 151-153)

2. *“Why don't you provide a hard set of criteria on your decision when to include or exclude a possibly biased paper? You state that "observational cross-sectional studies" will be included, but you do not provide the inclusion criteria when it comes to the quality of the respective studies.”*

**Response:** We appreciate the observation and recognize the importance of applying rigorous criteria to evaluate the quality of included studies. As outlined in our protocol, we utilize established risk-of-bias assessment tools to identify and mitigate potential sources of bias in the selected studies. However, as recommended by Cochrane (*“Therefore, review authors should systematically take into account* ***risk of bias in results of included studies*** *when interpreting the results of their review.”*), we did not exclude the studies based on their quality.

**Reference:** Boutron I, Page MJ, Higgins JPT, Altman DG, Lundh A, Hróbjartsson A. Chapter 7: Considering bias and conflicts of interest among the included studies [last updated August 2022]. In: Higgins JPT, Thomas J, Chandler J, Cumpston M, Li T, Page MJ, Welch VA (editors). *Cochrane Handbook for Systematic Reviews of Interventions* version 6.5. Cochrane, 2024. Available from [www.training.cochrane.org/handboo](http://www.training.cochrane.org/handbook)k

Also, we intend to do a sensitivity analysis removing studies with a high risk of bias from the meta-analysis and analysing the impact of the decision to include them.

*“A sensitivity analysis is a repeat of the primary analysis or meta-analysis in which alternative decisions or ranges of values are substituted for decisions that were arbitrary or unclear. For example, if the eligibility of some studies in the meta-analysis is dubious because they do not contain full details, sensitivity analysis may involve undertaking the meta-analysis twice: the first time including all studies and, second, including only those that are definitely known to be eligible.”*

**Reference:** Deeks JJ, Higgins JPT, Altman DG, McKenzie JE, Veroniki AA (editors). Chapter 10: Chapter 10: Analysing data and undertaking meta-analyses [last updated November 2024]. In: Higgins JPT, Thomas J, Chandler J, Cumpston M, Li T, Page MJ, Welch VA (editors). Cochrane Handbook for Systematic Reviews of Interventions version 6.5. Cochrane, 2024. Available from [www.training.cochrane.org/handbook](http://www.training.cochrane.org/handbook).

3. *"Discrepancies will be resolved with the third examiner" would not seem convincing. - With all these aspects in mind, please go to https://journals.plos.org/plosone/s/submission-guidelines#loc-systematic-reviews-and-meta-analyses. Remember that your study must follow the guidelines given with "Systematic reviews and meta-analyses".*

**Response:** We appreciate the observation and recognize the importance of a robust and transparent approach to resolving discrepancies between reviewers during the study selection and data extraction processes.

We have revised the text as follows:

“In the absence of consensus among the reviewers (A.B.O.M and L.R.H.), the final decision regarding the risk classification of the studies, whether low or high, will be made by a third reviewer (J.M.D.O).” (Page 9, line 202 to 204).

We based our methodology in:

*“Use (at least) two people working independently to determine whether each study meets the eligibility criteria. Ideally, screening of titles and abstracts to remove irrelevant reports should also be done in duplicate by two people working independently (although it is acceptable that this initial screening of titles and abstracts is undertaken by only one person). It is essential, however, that two people working independently are used to make a final determination as to whether each study considered possibly eligible after title/abstract screening meets the eligibility criteria based on the full text of the study report(s) (see* [*MECIR Box 4.6.c*](https://training.cochrane.org/handbook/current/chapter-04#_Ref531774841)*).”*

**Reference:** Lefebvre C, Glanville J, Briscoe S, Featherstone R, Littlewood A, Metzendorf M-I, Noel-Storr A, Paynter R, Rader T, Thomas J, Wieland LS. Chapter 4: Searching for and selecting studies [last updated September 2024]. In: Higgins JPT, Thomas J, Chandler J, Cumpston M, Li T, Page MJ, Welch VA (editors). *Cochrane Handbook for Systematic Reviews of Interventions* version 6.5. Cochrane, 2024. Available from [www.training.cochrane.org/handbook](https://training.cochrane.org/handbook).

**Reviewer #4:**

The authors have presented a study protocol for a systematic review on the prevalence of periodontitis among adolescents. The study protocol is interesting but has the following concerns:

1. *“It is unclear what this study protocol adds to the literature which cannot be comprehensively included in a Prospero registration.”*

**Response:** We appreciate the observation. While the PROSPERO registration provides an overview of the protocol, our study includes additional elements that justify the independent publication of the protocol. In particular, the protocol outlines the detailed methodology, inclusion and exclusion criteria, and risk-of-bias assessment procedures specific to our analysis, including a rigorous evaluation of cross-sectional observational studies. These aspects are important for the transparency and reproducibility of the research and are not fully addressed in a general PROSPERO registration.

Our protocol aims to contribute to the literature by providing a robust foundation for conducting the systematic review, detailing the specific aspects of our methodological approach and the criteria established to ensure the quality of the selected studies. Furthermore, the publication of a protocol makes it more accessible to the public, facilitating its retrieval, as seen, for instance, in searches on PubMed.

2. *“The authors state there are no systematic reviews with a similar scope. I would urge the authors to do a more comprehensive search. Articles such as: Catunda RQ, Levin L, Kornerup I, Gibson MP. Prevalence of Periodontitis in Young Populations: A Systematic Review. Oral Health Prev Dent. 2019;17(3):195-202. has not been cited nor discussed.”*

**Response:** We appreciate the suggestion and the recommendation of the article. We recognize the importance of conducting a comprehensive literature search. The study was identified subsequently, during the initial stages of the search. We appreciate the suggestion and inform that the article by Catunda et al. has been duly incorporated into the introduction.

CUnderstanding the prevalence of periodontitis in different populations is essential to guide prevention and treatment strategies, particularly in vulnerable groups, thereby helping to reduce the progression of the disease into adulthood. Given its global relevance and the regional and socioeconomic variations in the impact of periodontitis among adolescents, prevalence studies provide valuable data to support public health strategies [9]. The systematic review conducted by Catunda et al. (2019) investigated the prevalence of the disease in young individuals but presented significant methodological limitations, such as restricting studies to those published between 1999 and 2017, including case reports and case series, and the absence of a meta-analysis. To address these limitations, the present systematic review aims to estimate the prevalence of periodontitis in adolescents aged 13 to 18 years without restrictions regarding publication year, language, or country, focusing exclusively on cross-sectional studies. This study seeks to provide a precise and comprehensive estimate of this condition within this age group” (Page 4 and 5, line 95 to 108).

3. *“It is unclear how aggressive periodontitis and periodontitis will be analyzed/ segregated since majority of pre 2017 studies focussed on aggressive periodontitis as a separate and in many cases sole entity in adolescents. The authors do mention that they would segregate based on the definition but it maybe insufficient to fully capture the complexity by just definition.”*

**Response:** We appreciate the observation and acknowledge the complexity of differentiating aggressive periodontitis and periodontitis in adolescents, especially in studies prior to 2017. While we initially proposed segregation based on definitions, we agree that this approach may be insufficient to fully capture the complexity of these conditions.

To enhance the accuracy of our analysis, we intend to adopt a more detailed approach, considering the clinical characteristics and diagnostic criteria used in the studies, in addition to the formal definitions.

“Periodontitis should be diagnosed through clinical examination (alone or in combination with radiographic assessment), using clearly defined criteria for case definition, taking into account the clinical characteristics and diagnostic criteria applied in the studies, in addition to the formal definitions established.” (Page 6, line 129 to 124)

**Reference:** Heitz‐Mayfield, L.J. (2024). Conventional diagnostic criteria for periodontal diseases (plaque-induced gingivitis and periodontitis). *Periodontology 2000*.

4. *“The authors cite the GBD paper on periodontitis in the background section. The GBD study only reports on severe periodontitis. This needs to be clarified to avoid misinterpretations.”*

**Response:** We appreciate the observation. We acknowledge the importance of clarifying the focus of the GBD study. The introduction section has been revised accordingly.

“A study that collected data from 1990 to 2019, from the Global Burden of Disease 2019 study, reported that periodontitis was negatively associated with the level of socioeconomic development. Periodontitis was more frequent among those aged 55-59 years, but the incidence of periodontitis has shown an increasing trend among younger individuals.” (Page 3, line 68-72)

5. *“At the end of the background section the authors discuss aiming to find high risk groups and associated risk factors. The rest of the methods don't support this aim.”*

**Response:** We appreciate the thorough analysis. The methods section has been reviewed to ensure its full alignment with the study's objective, which has been appropriately revised.

The following presents the revised objective:

**“Sensitivity analysis**

A sensitivity analysis removing studies with a high risk of bias from the meta-analysis and analysing the impact of the decision to include them is planned. “

(Page 11, line 251-254)
