# Supplementary material for: Prevalence of periodontitis in adolescents: A systematic review protocol
Source: PLoS One. 2025 May 5;20(5):e0321993. doi: 10.1371/journal.pone.0321993 (PMC12052176; doi:10.1371/journal.pone.0321993)
Supplement: Attachment — Submitted filename: Response to Reviewers.docx [file pone.0321993.s006.docx]

Dear Dr.
Editor-in-Chief

PLOS ONE

We appreciate the opportunity to revise our article. We have carefully worked on incorporating the reviewers' suggestions to enhance the quality of the manuscript. Text modifications and additions were highlighted in yellow.

**Reviewer #1**:

1. *“With reference to adequacy of the literature search, please provide reasons why you think that including EMBASE, LILACS, MEDLINE (via PubMed), and Web of Science. would seem sufficient. There are other reliable databases, but why should the latter be excluded?”*

**Response:** Thank you for the feedback. The selection was based on their comprehensive coverage of biomedical and health sciences literature, ensuring a broad and relevant retrieval of studies. We have followed the orientations from the *Cochrane Handbook for Systematic Reviews.* As recommended by the *Handbook*, the search should be conducted in at least three databases. Additionally, gray literature will also be considered.

**Reference:** Lefebvre C, Glanville J, Briscoe S, Featherstone R, Littlewood A, Metzendorf M-I, Noel-Storr A, Paynter R, Rader T, Thomas J, Wieland LS. Chapter 4: Searching for and selecting studies [last updated September 2024]. In: Higgins JPT, Thomas J, Chandler J, Cumpston M, Li T, Page MJ, Welch VA (editors). *Cochrane Handbook for Systematic Reviews of Interventions* version 6.5. Cochrane, 2024. Available from [www.training.cochrane.org/handbook](https://training.cochrane.org/handbook).

2. *"One reviewer (A.B.O.M.) will collect data from the included studies, and a second reviewer (L.R.H.) will crosscheck them." What does "will crosscheck" mean? A simple and superficial crosscheck would not seem sufficient. Please note that best practice does require two review authors to INDEPENDENTLY determine (1) eligibility of studies for inclusion in systematic reviews and (2) data extraction. This involves checking the characteristics of a study against the elements of the research question.”*
3. *“Same with data extraction. The latter must be performed in duplicate. Please clarify.”*

**Response:** We appreciate your feedback. However, according to the *Cochrane Handbook for Systematic Reviews*, data extraction by a single author is not prohibited, similar to the study selection process. We tried to clarify this in the text as follows:

One reviewer (A.B.O.M.) will extract data from the included studies, while a second reviewer (L.R.H.) will thoroughly verify all extracted information by comparing the data extracted with the original data in the included studies. (Line 184 to 186, page 8).

**Reference:** Li T, Higgins JPT, Deeks JJ. Chapter 5: Collecting data [last updated October 2019]. In: Higgins JPT, Thomas J, Chandler J, Cumpston M, Li T, Page MJ, Welch VA (editors). *Cochrane Handbook for Systematic Reviews of Interventions* version 6.5. Cochrane, 2024. Available from [www.training.cochrane.org/handbook](http://www.training.cochrane.org/handbook).

4. *“Excluded and included studies must be separately considered. Excluded studies should be accounted for fully by the review authors, otherwise there is a risk that they remain invisible and the impact of their exclusion from the review is unknown.”*

**Response:** We appreciate the valuable comments. We would like to emphasize that, as required by the *Cochrane Handbook for Systematic Reviews*, this step will be performed. All excluded studies and reasons for exclusion will be properly documented and reported, ensuring transparency and clarity regarding the impact of their exclusion on the analysis process. “A Cochrane Review includes a list of excluded studies called ‘Characteristics of excluded studies’, detailing the specific reason for exclusion for any studies that a reader might plausibly expect to see among the included studies. This covers all studies that may, on the surface, appear to meet the eligibility criteria but which, on further inspection, do not. It also covers those that do not meet all of the criteria but are well known and likely to be thought relevant by some readers. By listing such studies as excluded and giving the primary reason for exclusion, the review authors can show that consideration has been given to these studies.”

We will rigorously follow the Cochrane guidelines to ensure the review's integrity and quality. We tried to clarify this in the text as follows:

“If important data for the review are missing or unclear, an attempt will be made to contact the study's corresponding author. The excluded studies with reasons for exclusion will be presented in the Appendix 2 of the final review.” (Line 180 to 182, page 8)

**Reference:**  Lefebvre C et al. Chapter 4: Searching for and selecting studies [last updated September 2024]. In: Higgins JPT, Thomas J, Chandler J, Cumpston M, Li T, Page MJ, Welch VA (editors). *Cochrane Handbook for Systematic Reviews of Interventions* version 6.5. Cochrane, 2024. Available from [www.training.cochrane.org/handbook](https://training.cochrane.org/handbook).

5. *Please revise for typos, see "reviewer’s authors".*

**Response:** Thank you for your comment. We revised the text accordingly.

6. *“With their paper, review authors must provide detail about research designs, study populations, interventions, comparators, and outcomes (including the sources of funding for the studies included in the review). The details should be sufficient for any appraisers to make a judgment about the extent to which the studies were appropriately chosen (in relation to the PICO). Please clarify.”*

**Response:** We appreciate your inquiry and recognize the significance of providing comprehensive details on the studies included in the review. This ensures transparency and facilitates a critical evaluation of the appropriateness of the selection process concerning the PICO framework. To enhance clarity, we have revised the text as follows:

“The data will be stored in a data extraction form that will be developed using a spreadsheet program (Microsoft Excel; Microsoft Corporation, Redmond, WA, USA). If disagreements arise, a third reviewer (J.M.D.O.) will be contacted for a final decision. The collected information will include the author, study design, publication year country, patient characteristics (sample size, sex, and age), clinical characteristics (diagnostic methods, definition of periodontitis cases, case of periodontitis by sex, periodontal criteria confidence, periodontal examination protocol, periodontitis/healthy or gingivitis and extension/distribution), and outcomes (prevalence rates).” (Line 189 and 194, page 9)

7. *“Same with RoB. Please assure to account for RoB in primary studies when interpreting/discussing the results of the review.”*

**Response:** We sincerely appreciate the thoughtful feedback provided. We will conduct a sensitivity analysis to assess the impact of bias risk by excluding studies with high risk of bias from the meta-analysis. This approach aims to evaluate the robustness of the results. In the discussion section, we will address the impact of studies with high risk of bias through this sensitivity analysis, which appropriately responds to the raised question. The revised paragraph is presented below:

“A sensitivity analysis removing studies with a high risk of bias from the meta-analysis and analysing the impact of the decision to include them is planned”. (Line 258 to 259, page 12)

8. *“Describe where all data underlying the findings will be made available when the study is complete.”*

**Response:** Thank you for your suggestion. We will use the Open Science Framework (OSF) platform as a repository to make the original data available, ensuring transparency and accessibility.

This will encompass adjustments to the inclusion and exclusion criteria, along with the justification for each change and an assessment of its impact on the review's results. We will use the Open Science Framework (OSF) platform as a repository to make the original data available, ensuring transparency and accessibility. (Line 164 to 166, page 8)

9. *“Ensure that your systematic review design provides an accurate and comprehensive summary of the results of the available studies that address the question of interest. Ensure reporting the key sequential steps in the conduct of a systematic review, to ensure future high quality assessment of your SR.”*

**Response:** We sincerely appreciate your valuable suggestion. We followed the PRISMA-P guidelines to conduct this protocol. The systematic review will be reported in accordance with the PRISMA 2020 recommendations.
